# Significant differences in knee kinematics of healthy subjects with high and low anterior tibial laxity

**DOI:** 10.3389/fbioe.2024.1514516

**Published:** 2024-12-09

**Authors:** Shiyang Chen, Shaohua Chen, Qingyang Kang, Fangzheng Lin, Shuting Zheng, Xixi Liu, Chunhong Guo, Yongjin Li, Dingkun Lin, Xiaolong Zeng

**Affiliations:** ^1^ The Second Clinical Medical College, Guangzhou University of Chinese Medicine, Guangzhou, China; ^2^ Orthopedics Department, The Second Affiliated Hospital of Guangzhou University of Chinese Medicine (Guangdong Provincial Hospital of Chinese Medicine), Guangzhou, China; ^3^ Wangjing Hospital of China Academy of Chinese Medical Sciences, Beijing, China; ^4^ Key Laboratory of Beijing of TCM Bone Setting, Beijing, China; ^5^ Chinese Medicine Guangdong Laboratory, Zhuhai, China; ^6^ Guangdong Provincial Key Laboratory of Clinical Research on Traditional Chinese Medicine Syndrome, Guangzhou, China

**Keywords:** knee, anterior tibial laxity, knee kinematics, kinematic alterations, dynamic stability

## Abstract

**Background:**

Anterior tibial laxity is considered to be a risk factor for knee injuries, including anterior cruciate ligament ruptures. The anterior cruciate ligament reconstruction also aims to restore anterior tibial laxity. While anterior tibial laxity is considered to be linked to dynamic knee stability, the mechanisms connecting anterior tibial laxity to these stability issues are not fully understood. The purpose of this study was to investigate the kinematic alterations between different anterior tibial laxity in healthy subjects. We hypothesized that anterior tibial laxity affects the anteroposterior tibial displacement during dynamic movements.

**Methods:**

This study involved thirty-five healthy subjects. There were twenty males and fifteen females with an average age of 18.91 ± 0.78 years. Their knees were categorized into “Tight” (the smallest 50%) and “Lax” (the largest 50%) groups based on anterior tibial laxity measurements using a Kneelax3 arthrometer. Kinematic data were collected using a three-dimensional motion capture system when they performed level walking, upslope walking, and vertical jumping. The knee kinematics were recorded for statistical analysis. We used independent sample t-tests to analyze key kinematic differences between groups.

**Results:**

The “Lax” group exhibited increased posterior tibial translation during upslope walking (5.4 ± 2.22 mm at swing max flexion, *p* = 0.018) and vertical jumping (8.5 ± 2.78 mm at propulsion max flexion, *p* = 0.003; 7.6 ± 3.17 mm at landing max flexion, *p* = 0.019) than the “Tight” group. Significant differences in tibial internal rotation were observed during initial contact of the gait cycle of level walking (1.9° ± 0.95°, *p* = 0.049) and upslope walking (2.1° ± 1.03°, *p* = 0.041) in the “Lax” group compared to the “Tight” group. No significant differences in adduction/abduction or medial/lateral tibial translation were found between groups.

**Conclusion:**

The study revealed that high anterior tibial laxity resulted in increased posterior tibial translation and tibial internal rotation. High anterior tibial laxity resulted in dynamic instability of knees during motions, especially in high-demanding activities like upslope or vertical jumping. However, further research is needed to explore the clinical functional effects of knee laxity.

## 1 Introduction

Anterior tibial laxity (ATL) refers to the anterior translation of the tibia relative to the femur. It affects knee stability and is primarily controlled by the anterior cruciate ligament (ACL) (Zlotnicki et al., 2016; Nishizawa and Tashman, 2017). Previous studies have identified ATL as a risk factor for knee injuries, including ACL ruptures (Wetters et al., 2016). In addition, studies have shown that in cases of primary ACL deficiency (ACLD), increased anterior tibial translation is associated with meniscus or cartilage damage, and early-onset knee osteoarthritis (Sommerfeldt et al., 2018; Li et al., 2020). It is believed that anterior cruciate ligament reconstruction (ACLR) aims to restore normal ATL to regain proper joint kinematics and restore knee stability after ACL injury (Amano et al., 2016; Park et al., 2021). These emphasize the significance of ATL in the context of knee joint mechanics.

Previous studies have investigated the relationship between static anterior laxity and dynamic knee stability with conflicting results. Static anterior knee laxity has been found to be correlated with knee kinematics during specific activities. Specifically, Keizer et al. (2020) found a weak negative correlation (*r* = −0.47, *p* = 0.028) between passive and dynamic anterior tibia translation during jump landing in healthy individuals. In contrast, a positive correlation between passive ATL and anterior-posterior tibial translation has been reported among recreational athletes during vertical jumping and ACLD patients during walking (Torry et al., 2011; Boeth et al., 2013). Additionally, Sato et al. (2013) observed that maximum tibial internal rotation during side-step cutting negatively correlated with static anterior tibial translation in seven subjects with unilateral ACLRs, suggesting that greater laxity could influence rotational knee kinematics. However, several studies have found no significant correlations between static knee laxities and dynamic kinematics, emphasizing that measurements of static laxity alone are insufficient for assessing functional status after ACL injury (Musahl et al., 2012; Thorhauer et al., 2014; Tagesson et al., 2015). Sonesson and Kvist (Sonesson and Kvist, 2017) have shown that anterior tibial laxity was not associated with dynamic tibial translation when comparing dynamic and static tibial translation in the ACL-deficient knees at 2- to 5-year follow-up with directly after rehabilitation.

The results of previous studies have shown inconsistencies since the study population is mostly ACLD/ACLR patients. The knee status was affected by the disease, the different measurements and analysis methods used in the study, and the different types of exercise selected. The influence of these confounding factors cannot be ruled out and the exact mechanism by which anterior tibial laxity might affect knee stability is not fully understood. Consequently, studies are needed to examine the relationship between ATL and dynamic stability in healthy subjects. Investigating this relationship can provide insights into the understanding of ATL in the natural variability of knee mechanics and inform the development of more targeted interventions to enhance knee stability (Nishizawa and Tashman, 2017; Lutz et al., 2021). The study aimed to compare the kinematic characteristics of knee joints in healthy subjects with high and low ATL under different exercise conditions. We hypothesized that ATL significantly affects the anteroposterior tibial displacement during dynamic movements.

## 2 Methods

### 2.1 Subjects

The study was approved by the Ethics Committee of Guangdong Provincial Hospital of Chinese Medicine (ZE 2024-101-01). Written consent was obtained from all the participants after they were fully informed of the experiment. The subjects were recruited if: (1) no history of major injury or surgery in the knees; (2) no musculoskeletal or neurological diseases affecting motor function; and (3) no vigorous exercise within 24 h before the experiment. Our study enrolled 35 healthy subjects (20 males and 15 females) between January and April 2024. The participants’ demographic data were the age of 18.91 ± 0.78 years and the BMI of 21.91 ± 3.28 kg/m^2^. They had an International Physical Activity Questionnaire median (range) total score of 1,710 (4,491). The majority of participants had moderate levels of physical activity, with 5.7%, 82.9%, and 11.4% being categorized as low, moderate, and high activity levels, respectively. According to the results in anterior tibial laxity, the 70 knees from 35 participants were divided into two groups. The “Tight” group (n = 35) consisted of knees with the smallest 50% of ATL, while the “Lax” group (n = 35) consisted of knees with the largest 50% of ATL (the “Tight” group vs the “Lax” group; 4.02 ± 0.96 mm vs 7.28 ± 1.34 mm, *p* < 0.001).

### 2.2 Devices and experiment procedures

Knee anterior tibial laxity was measured using a Kneelax3 arthrometer (Monitored Rehab Systems, Haarlem, The Netherlands). The Kneelax3 arthrometer has been reported to be frequently used for the studies investigating the ligament laxity with a measurement accuracy of 0.1 mm (Shi et al., 2013). Participants’ knee movements were recorded using a three-dimensional motion capture gait system (Opti_Knee, Innomotion Inc., Shanghai, China) to obtain six-degree-of-freedom (6DOF) knee kinematics data (Zhang et al., 2015; Wang S. et al., 2021). The gait system included a navigation stereo infrared tracking device (NDI Polaris Spectra; Northern Digital Inc.), a high-speed optical camera (Basler aca640–90uc; Basler AG), two sets of markers, a handheld digital probe, and a level motorized bi-directional and adjustable-sloped treadmill. The system sampled data at a rate of 120 Hz. It has been previously described in studies and is known for its high accuracy with a reported root mean square accuracy of 0.3 mm (Elfring et al., 2010) and a repeatability of less than 1.3° in rotation and 0.9 mm in translation (Zhang et al., 2016).

To determine their eligibility for the experiment, each subject underwent an initial check by an orthopedic surgeon (FL) skilled in physical examination and scale assessment. They first completed the International Physical Activity Questionnaire and were then asked about any history of injuries, followed by a clinical knee examination. The same orthopedic surgeon (FL), who was skilled in the use of Kneelax3, measured the ATL of each subject. On an examination table, each subject was positioned supine with the knee in approximately 30 degrees of flexion. The laxity of both knees was measured as anterior tibial translation of the femur with an anterior force of 132N.

**FIGURE 1:**
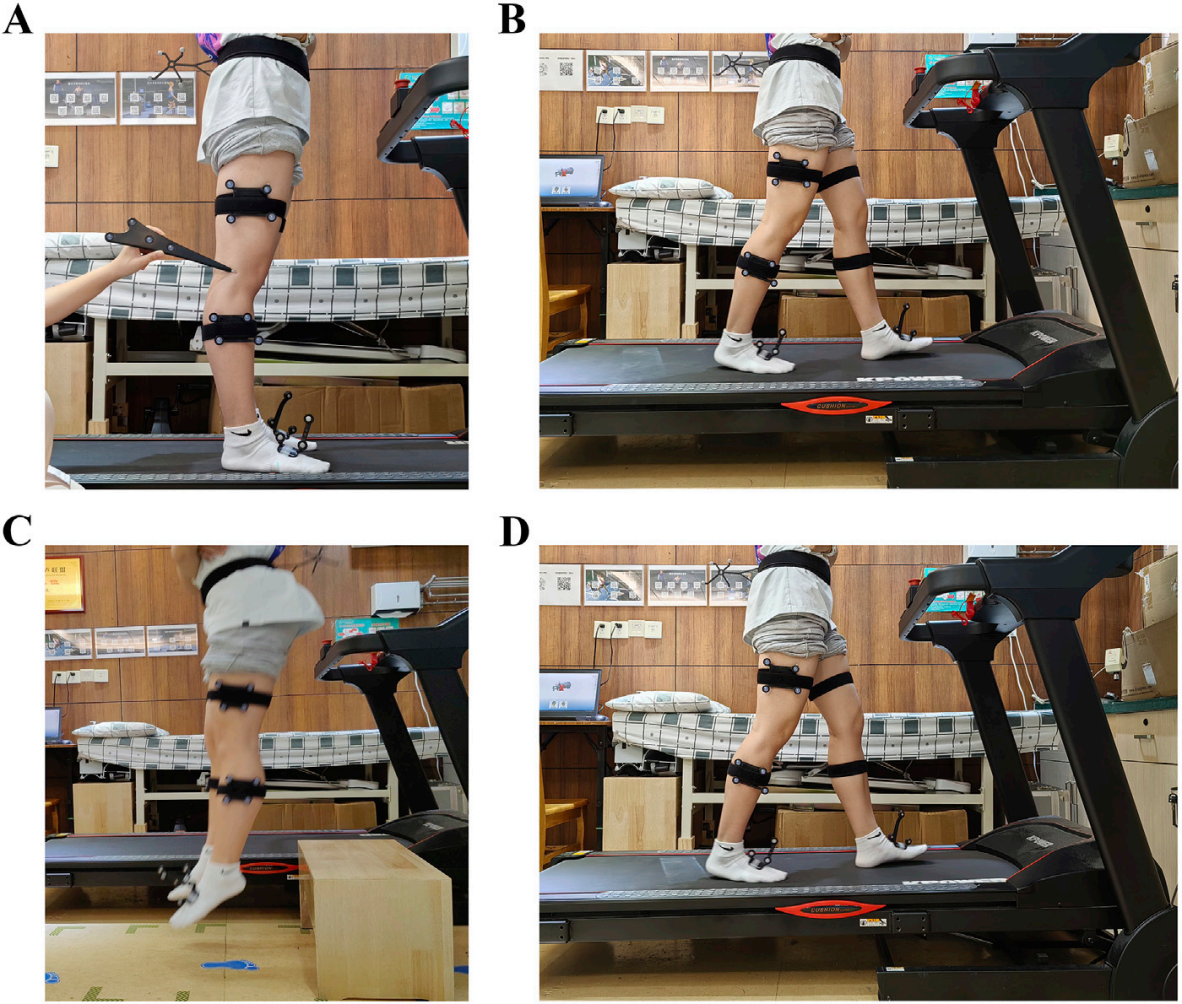
**(A)** The marker sets were attached to the thigh and shin, and a handheld digital probe was used to identify patient-specific bony landmarks. **(B)** The level walking test. **(C)** The vertical jumping test. **(D)** The upslope walking test.

Participants then performed three experimental tasks—level walking, upslope walking, and vertical jumping—to collect kinematic data. These three tasks are relevant to knee mechanics and common in daily and sports activities (Õunpuu, 1994; Kuster et al., 1995; Ryan et al., 2006). Kinematic data collection was managed by the authors (ShiC and QK), who were blinded to the participants’ clinical characteristics during the recruitment and ATL measurements. Both authors are highly trained and experienced in gait system operations. Before performing the test system calibration, the participants were instructed to exposed their lower limbs and maintain a neutral standing position with their arms akimbo to avoid blocking the markers. The marker sets were attached to each participant’s thigh and shin while in a standing position, and a handheld digital probe was used to identify patient-specific bony landmarks, including the great trochanter, medial and lateral epicondyles, medial and lateral tibial plateaus, and medial and lateral malleoli (Figure 1A). The neutral standing position was also used as a zero reference. Following the identification procedure, customized three-dimensional tibiofemoral coordinate systems were automatically created by the system’s software.

Before data collection, each participant walked on a level motorized bi-directional treadmill for 5∼10 min to adapt to the treadmill. Once ready, the participants informed the test operator, and the walking test began with the walking speed of 3.0 km/h (Figure 1B). After 10 s of stable walking, knee joint velocity data was collected for about 15 s (15 walking gait cycles) for both knee joints under the same experimental conditions. Participants performed upslope walking on the treadmill with a 5% slope (Figure 1D). Data collection was set at 15-second intervals, equivalent to 15 upslope gait cycles. For the vertical jumping, participants were instructed to drop from a 30 cm box and land on both feet simultaneously with hands on each side of their hips (Figure 1C). The vertical jumping was repeated three times for both knee joints to obtain effective kinematic knee data. There was a 5-minute break between the two experiment tasks for each participant to avoid fatigue effects and the influence of the previous test. The system automatically averaged the kinematics from all the test cycles into an averaged cycle, which included 101 data points. The knee kinematic data were extracted for statistical analysis.

### 2.3 Statistical analysis

The time points for both level and upslope walking we analyzed were initial contact (0% of the gait cycle), midstance (31% of the gait cycle), and swing phase maximum flexion (76% of the gait cycle). These time points are significant because they correspond to critical phases of the gait cycle, each reflecting different biomechanical demands and kinematic behaviors. For vertical jumping, the selected time points (propulsion stance, propulsion maximum flexion, landing maximum flexion, and landing stance) are similarly significant for understanding knee mechanics under different load conditions (Jiang, 2017; Gait and Balance Academy, 2018).

The mean and standard deviation were calculated for each variable of the participants’ “Tight” and “Lax” legs during 3 different experiment tasks. Before conducting independent t-tests, the normal distribution of the participants’ data was verified using the Shapiro-Wilk test. Independent sample t-tests were then used to determine the significant differences between the “Tight” and “Lax” groups in each biomechanical variable (*p* < 0.05). All the statistical procedures were conducted using IBM SPSS Statistical Software (Version 27.0; SPSS, Inc., Chicago, IL, United States).

## 3 Results

**FIGURE 2:**
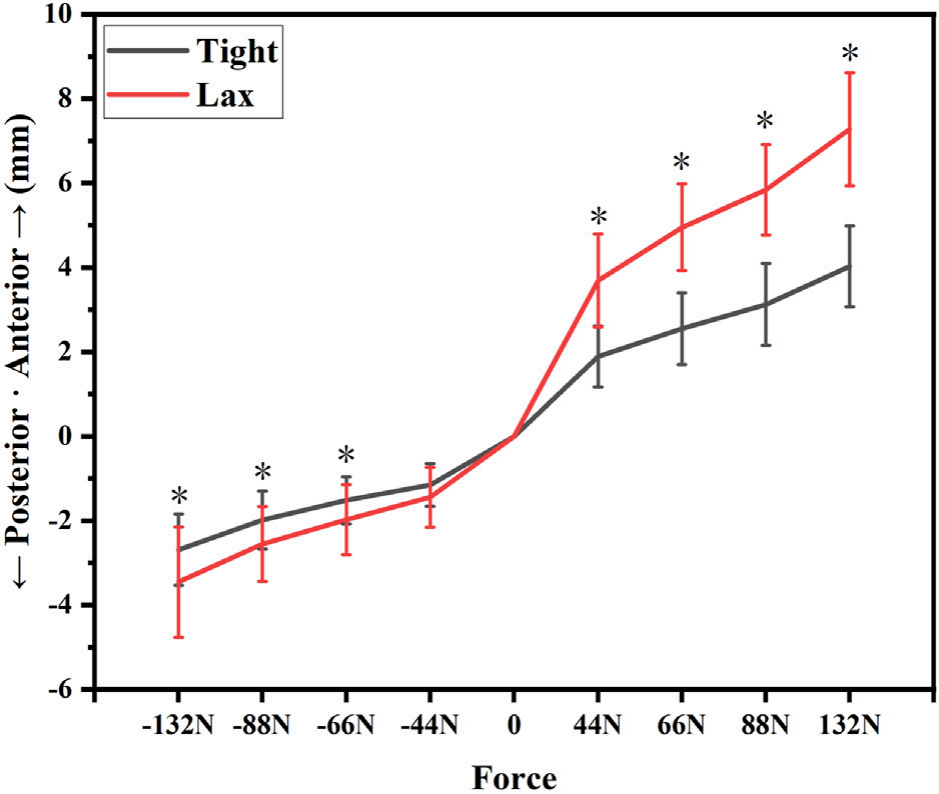
The anterior-posterior tibial laxity curves of the “Tight” group and the “Lax” group. Notes: * indicates significant differences between the “Tight” group and the “Lax” one (*p* < 0.05).

**FIGURE 3:**
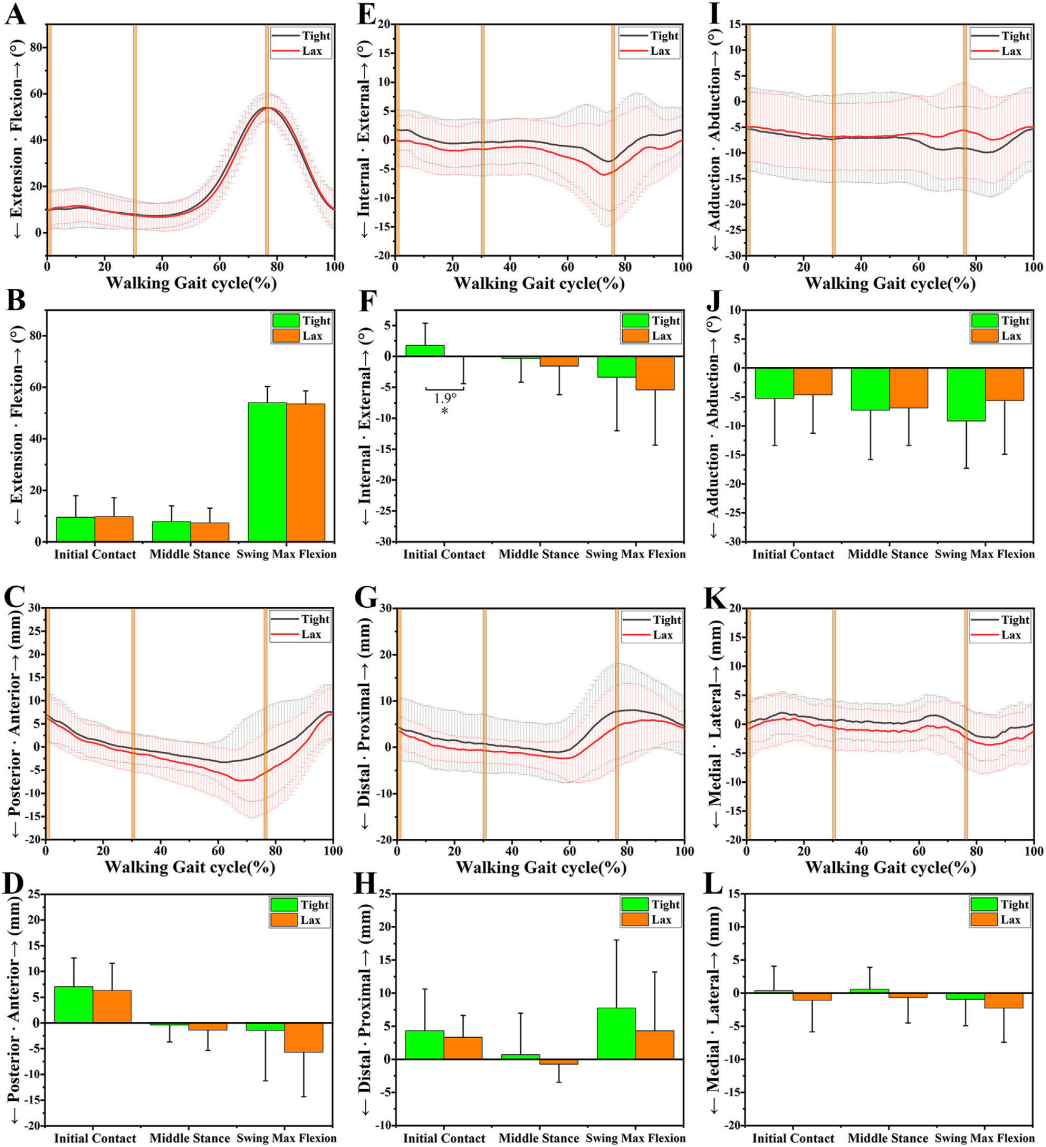
The 6DOF knee kinematic alterations between the “Tight” and “Lax” groups during level walking. **(A-B)**, A shows the flexion angle alterations between two groups during a walking gait cycle and B shows their comparison at three primary time points. **(C-D)**, C shows the anteroposterior tibial translation alterations between two groups during a walking gait cycle and D shows their comparison at three primary time points. **(E-F)**, E shows the internal/external tibial rotation angle alterations between two groups during a walking gait cycle and F shows their comparison at three primary time points. **(G-H)**, G shows the distal/proximal tibial translation alterations between two groups during a walking gait cycle and H shows their comparison at three primary time points. **(I-J)**, I shows the adduction/abduction angle alterations between two groups during a walking gait cycle and J shows their comparison at three primary time points. **(K-L)**, K shows the medial/lateral tibial translation alterations between two groups during a walking gait cycle and L shows their comparison at three primary time points. Notes: * indicates significant kinematic differences between the “Tight” group and the “Lax” one (*p* < 0.05).

**FIGURE 4:**
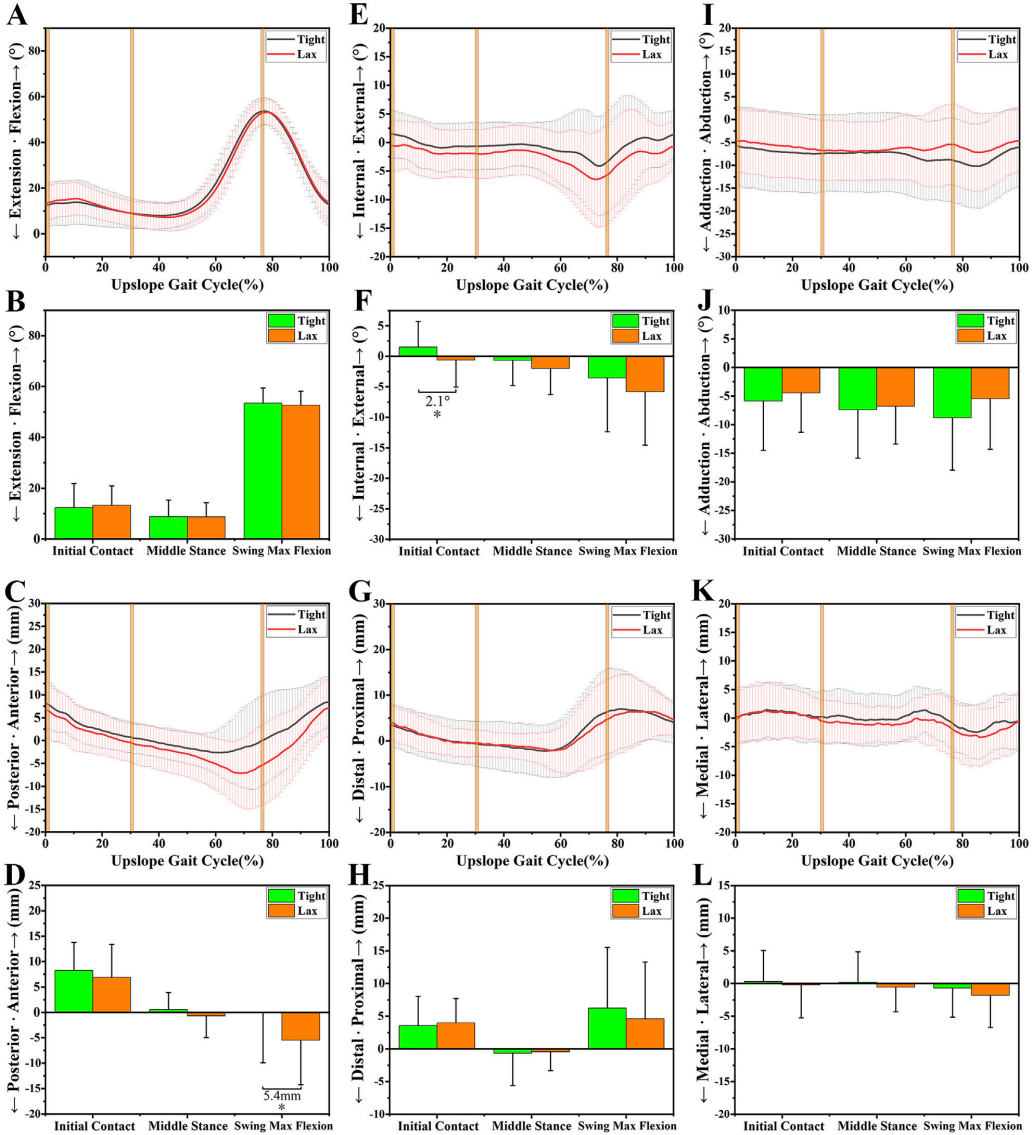
The 6DOF knee kinematic alterations between the “Tight” and “Lax” groups during upslope walking. **(A-B)**, A shows the flexion angle alterations between two groups during an upslope gait cycle and B shows their comparison at three primary time points. **(C-D)**, C shows the anteroposterior tibial translation alterations between two groups during an upslope gait cycle and D shows their comparison at three primary time points. **(E-F)**, E shows the internal/external tibial rotation angle alterations between two groups during an upslope gait cycle and F shows their comparison at three primary time points. **(G-H)**, G shows the distal/proximal tibial translation alterations between two groups during an upslope gait cycle and H shows their comparison at three primary time points. **(I-J)**, I shows the adduction/abduction angle alterations between two groups during an upslope gait cycle and J shows their comparison at three primary time points. **(K-L)**, K shows the medial/lateral tibial translation alterations between two groups during an upslope gait cycle and L shows their comparison at three primary time points. Notes: * indicates significant kinematic differences between the “Tight” group and the “Lax” one (*p* < 0.05).

**FIGURE 5:**
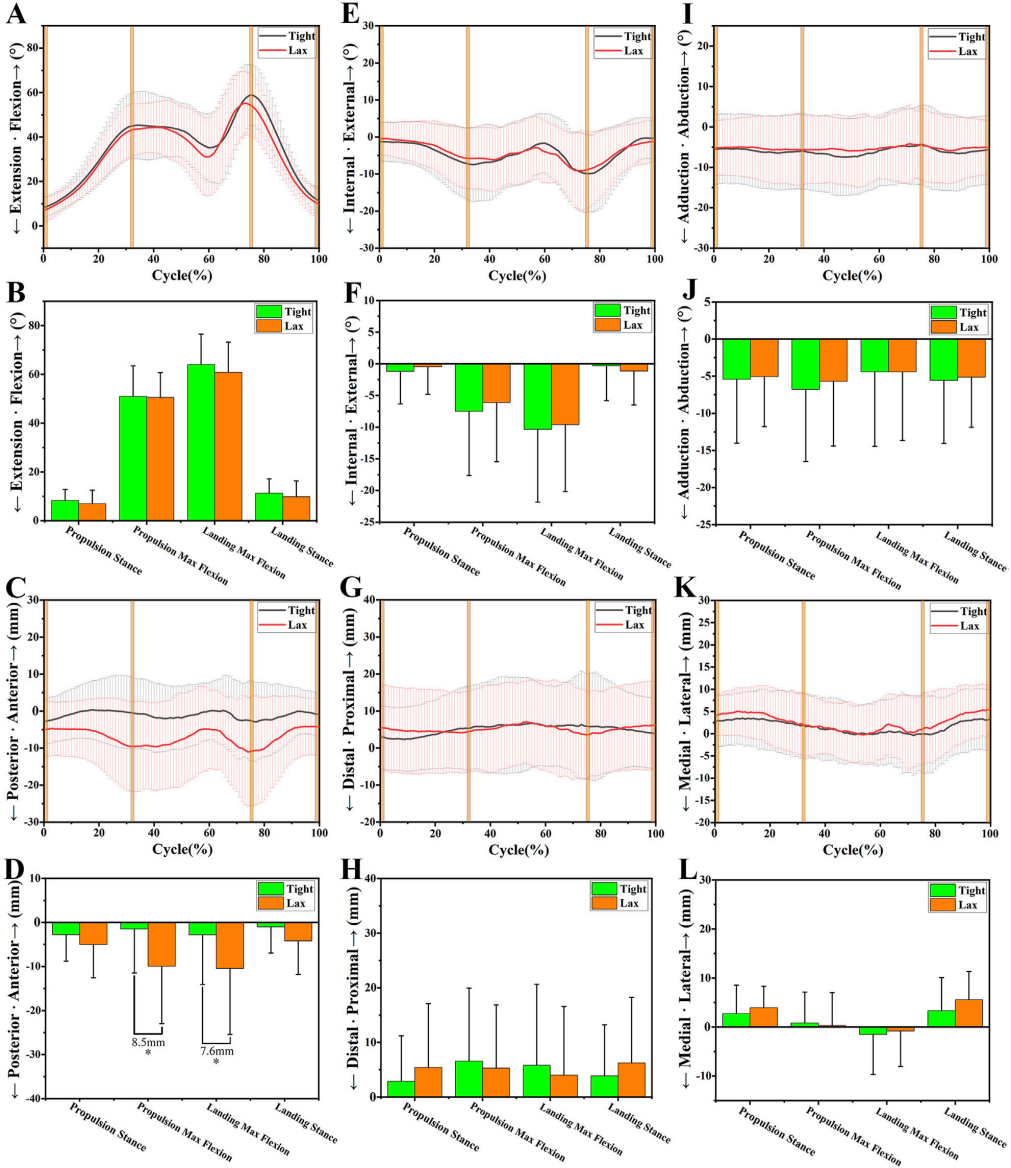
The 6DOF knee kinematic alterations between the “Tight” and “Lax” groups during vertical jumping. **(A-B)**, A shows the flexion angle alterations between two groups during a vertical jumping cycle and B shows their comparison at four primary time points. **(C-D)**, C shows the anteroposterior tibial translation alterations between two groups during a vertical jumping cycle and D shows their comparison at four primary time points. **(E-F)**, E shows the internal/external tibial rotation angle alterations between two groups during a vertical jumping cycle and F shows their comparison at four primary time points. **(G-H)**, G shows the distal/proximal tibial translation alterations between two groups during a vertical jumping cycle and H shows their comparison at four primary time points. **(I-J)**, I shows the adduction/abduction angle alterations between two groups during a vertical jumping cycle and J shows their comparison at four primary time points. **(K-L)**, K shows the medial/lateral tibial translation alterations between two groups during a vertical jumping cycle and L shows their comparison at four primary time points. Notes: * indicates significant kinematic differences between the “Tight” group and the “Lax” one (*p* < 0.05).

**FIGURE 6:**
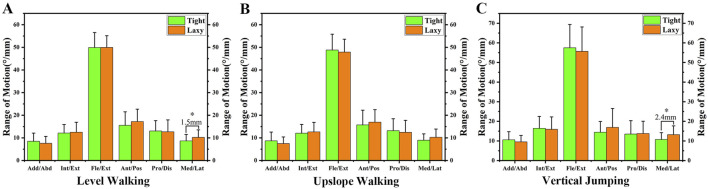
**(A)** The ROM of knee kinematics during level walking. **(B)** The ROM of knee kinematics during upslope walking. **(C)** The ROM of knee kinematics during vertical jumping. Notes: * indicates significant differences between the “Tight” group and the “Lax” one (*p* < 0.05).

The anterior-posterior tibial laxity curves of the “Tight” group and the “Lax” group are shown in Figure 2. The effects of knee anterior tibial laxity on 6DOF knee kinematics during three different experiment tasks (level walking, upslope walking, and vertical jumping) are summarized in Figures 3–6.

### 3.1 Sagittal plane

No significant kinematic difference in the sagittal plane’s kinematics parameters was shown at level walking (Figures 3A–D). However, during upslope walking, the “Lax” group showed a greater posterior tibial translation of 5.4 ± 2.22 mm than the “Tight” group at swing max flexion (*p* = 0.018; Figures 4C, D). Additionally, we found that the “Lax” group had increased posterior tibial translation than the “Tight” group at propulsion max flexion (8.5 ± 2.78 mm, *p* = 0.003) and landing max flexion (7.6 ± 3.17 mm, *p* = 0.019) in the vertical jumping cycle (Figures 5C, D).

### 3.2 Transverse plane

As for the transverse plane, significant kinematic differences were found during level walking and upslope walking, but not during vertical jumping. Compared with the “Tight” group, the “Lax” group showed a significant increase in internal rotation of 1.9° ± 0.95° (*p* = 0.049) at initial contact (IC) during level walking (Figures 3E, F). During upslope walking, the knee internal rotation angle of the “Lax” group was found to be 2.1° ± 1.03° (*p* = 0.041) larger than that of the “Tight” group during IC (Figures 4E, F).

### 3.3 Coronal plane

In the coronal plane, there was no significant difference in adduction/abduction and medial/lateral tibial translation between the knees with high and low ATL during different experiment tasks (Figures 3I–L, 4I–L; Figures 5I–L). However, during level walking, the “Lax” group showed a greater range of motion (ROM) for medial/lateral tibial translation compared to the “Tight” group (1.5 ± 0.74 mm, *p* = 0.041). The same significant difference was observed during vertical jumping (2.4 ± 0.96 mm, *p* = 0.013; Figure 6).

## 4 Discussion

### 4.1 Main finding of the study

This study investigated the knee joint performance of healthy subjects under various exercise conditions, differentiating between knees with high ATL (the “Lax” group) and those with low ATL (the “Tight” group). The findings indicated significant kinematic differences in the sagittal and transverse planes between these groups. During upslope walking and vertical jumping, the “Lax” group exhibited increased posterior tibial translation than the “Tight” group. The “Lax” group demonstrated greater external rotation of the tibia in both level and upslope walking. In addition, the “Lax” group showed a larger ROM in medial/lateral tibial translation during level walking and vertical jumping. These results may suggest that individuals with higher ATL experience less knee dynamic stability. Understanding these kinematic alterations is critical to develop targeted rehabilitation and injury prevention strategies for individuals with increased ATL or recovering from ACL injuries.

### 4.2 Sagittal plane

The findings of the sagittal plane confirmed our hypothesis that there would be significant kinematic alterations of the tibial anteroposterior position between different anterior tibial laxity during high-demand motions. During upslope walking, the posterior tibial translation increased in the “Lax” group compared to the “Tight” group at swing max flexion. In the vertical jumping cycle, the “Lax” group had increased posterior tibial translation than the “Tight” group during propulsion max flexion and landing max flexion. Our results are conflict with the findings of Torry et al. (2011). They found a strong relationship between passive anterior knee laxity measured via KT1000 and peak anterior tibial translation measured by biplane fluoroscopy system in healthy subjects performing vertical jumping. However, our findings align with Torry’s conclusions to some extent. This increased posterior tibial translation at knee flexion in our study may be attributed to the initial coordinate calibration of the three-dimensional motion capture gait system. At the time of calibration, the subject is in a standing position and exhibits the natural anterior tibial translation due to the effects of posterior tibial slope or other factors (Giffin et al., 2004; Wu et al., 2010; Dejour et al., 2019), whereas the system assumes that it is neutral and the translation displacement of the tibia is zero. So, when the knee is flexed, the contraction of the hamstrings can further exacerbate posterior tibial movement, particularly in individuals with high ATL. For patients with ACLD, Nishizawa and Tashman (Nishizawa and Tashman, 2017) have found no significant correlations between anterior tibial laxity and dynamic tibial translation. Over all, along with our findings, the high anterior tibial laxity is possibly related to anteroposterior tibial translation in healthy subjects, but further studies are needed to determine this relationship in ACLD/ACLR patients.

### 4.3 Transverse plane

In addition to anteroposterior tibial translation, the “Lax” group showed greater internal tibial rotation than the “Tight” group during level and upslope walking. This internal rotation may be due to altered knee mechanics and may be exacerbated by increased ACL laxity, which can compromise knee stability (Hazari et al., 2021). This observation aligns with the findings of Sato et al. (2013), who reported that greater laxity could influence rotational knee kinematics. They found that maximum tibial internal rotation during side-step cutting negatively correlated with static anterior tibial translation. Nevertheless, the exact mechanism of anterior tibial laxity affecting tibial rotation is not fully understood and is needed for further study.

### 4.4 Coronal plane

Different experimental tasks showed no significant differences between knees with high and low anterior tibial laxity in the coronal plane. Compared to the “Tight” group, the “Lax” group exhibited a larger ROM in medial/lateral tibial translation during level walking and vertical jumping. The increased ROM suggests the potential implications of high ATL for coronal plane knee stability. There is little literature on coronal plane stability in patients with anterior tibial laxity, and the literature usually focuses on the sagittal and transverse planes. In the research of Krosshaug et al. (Krosshaug et al., 2016), the medial knee displacement was considered as a screening test for predicting ACL injury, but ROC analysis indicated a poor combined sensitivity and specificity. Greater laxity could lead to increased variations in varus or valgus alignment during dynamic activities, thus affecting overall knee mechanics (Ortiz et al., 2014; Pinskerova and Vavrik, 2020; Hazari et al., 2021). Therefore, understanding the contribution of the coronal plane is critical as it provides insight into potential compensatory mechanisms or alignment issues that may arise due to increased anterior tibial laxity (Pinskerova and Vavrik, 2020).

### 4.5 Implications for dynamic stability

These kinematic alterations have significant implications for dynamic stability and injury risk. Individuals with higher ATL have greater tibial translation and rotation, which can lead to inefficient movement patterns and increased energy expenditure. This instability is particularly problematic during high-demand activities such as upslope walking and vertical jumping, where precise control of knee motion is critical for performance and injury prevention (Musahl et al., 2012; Migliorini et al., 2023). It may suggest that individuals with higher ATL could strengthen the quadriceps muscles to reduce posterior tibial displacement during exercise. Under normal circumstances, the ACL, other ligaments, and surrounding muscles work together to limit the front-to-back and inside-out movement of the tibia relative to the femur. When knee joint laxity increases, these limiting mechanisms can still function to a certain extent, so although the anteroposterior displacement and internal/external displacement increase, they will not reach pathological levels (Abulhasan and Grey, 2017). However, the consistent kinematic performance of the “Tight” group across various movements underscores the importance of maintaining adequate joint stiffness. This stability can be attributed to better neuromuscular control and stronger proprioceptive feedback mechanisms, which help maintain joint integrity and reduce the risk of injury (Hughes et al., 2019; Shagawa et al., 2021; Wang K. et al., 2021; Patel et al., 2023).

### 4.6 Limitations

This study has some limitations that need to be noticed. First, recruiting only healthy participants may restrict generalizability, though this selection helps reduce confounding factors from knee diseases. Future studies will aim to include a more diverse patient population to broaden the applicability of our findings. Second, the Opti_Knee gait system is susceptible to soft tissue artifacts, thereby the captured data may be compromised. However, two marker sets were attached to the distal third of the thigh and shank, which have been shown to minimize soft tissue artifact as much as possible (Akbarshahi et al., 2010; Schache et al., 2011). Further, we treated each knee as independent in this study, reflecting clinical observations of varied knee laxity between an individual’s knees. While this aligns with clinical realities, it might raise a statistical consideration regarding optimal analysis approaches. Finally, the clinical significance of our findings may be uncertain without a defined minimal meaningful difference for ATL and kinematic stability criteria. Further research is needed to establish clinically significant kinematic thresholds for knee laxity.

## 5 Conclusion

The study’s findings indicate that individuals with high anterior tibial laxity have less kinematic stability, particularly in the sagittal and transverse planes. While this instability could lead to an increased risk of injuries, such as ACL tears or meniscal damage, especially during high-impact activities, these inferences remain speculative. Future research should aim to provide a more comprehensive analysis, incorporating various dynamic conditions and a broader range of kinematic parameters, to better understand the functional implications of knee laxity and its potential association with injury risk.

## Data Availability

The raw data supporting the conclusions of this article will be made available by the authors, upon reasonable request.
